# Severe penile injury due to condom catheter fixed by a rubber band: A case report

**DOI:** 10.1016/j.ijscr.2019.10.009

**Published:** 2019-10-15

**Authors:** Selim Zaghbib, Marouene Chakroun, Ahmed Saadi, Hamza Boussaffa, Abderrazak Bouzouita, Amine Derouiche, Mohamed Riadh Ben Slama, Haroun Ayed, Mohamed Chebil

**Affiliations:** Departement of Urology, Charles Nicolle Hospital, Tunis, Tunisia

**Keywords:** Penile gangrene, Condom catheter, Strangulation

## Abstract

**Introduction:**

Condom catheters are considered as a safe tool in the management of male urinary incontinence, yet complications may occur, especially in debilitated patients, with psychiatric disorders.

**Presentation of case:**

A 58-year-old paraplegic man, suffering from schizophrenia, who had been using condom urinary catheters, was referred to our department for “penile injury”. Interrogation revealed that he was placing a rubber band tightly around his penis to maintain the condom. Examination showed a 4 cm defect interesting the proximal portion of the penis including the corpus spongiosum and the urethra. A surgical repair was performed, consisting in covering the defect using the surrounding tissue. Unfortunately, the patient developed penile gangrene four weeks after surgery. A partial penectomy was performed.

**Discussion:**

Condom catheters are known as a safe tool to manage urinary incontinence in male patients, providing an adequate care. Long-term use may result in complications including urinary tract infection (40%) and skin injuries ranging from inflammation to gangrene. Gangrene, secondary to strangulation of the penis, is however uncommon. These complications are more common in debilitated patients with poor medical care or psychiatric disorders. In case of necrosis, debridement should be performed, which may result in a partial or a total penectomy if necessary.

**Conclusion:**

Condom catheter is frequently used to manage male urinary incontinence but it should not be used carelessly or overlooked as it can cause severe complications such as penile strangulation and penile gangrene.

## Highlights

• Condom catheter is frequently used to manage male urinary incontinence but should not be used carelessly or overlooked.
• Even if they are rare, penile strangulation and gangrene may occur and are severe complications.
• Treatment is based on debridement, broad-spectrum antibiotics and skin grafting. It may result in partial or total penectomy.
• Proper care and routine maintenance of condom catheters are mandatory in order to prevent devastating complications.
• Appropriate care is necessary, especially in debilitated and psychiatric populations.

## 1 Introduction

Condom catheters are widely applied in bedridden and incontinent patients [1]. They are considered to be safe providing adequate care after their placement. Nevertheless, poor medical care of debilitated patients, especially when associated with psychiatric disorders, can lead to severe complications such as strangulation which is a rare condition [1]. Indeed, only few cases have been reported in the literature [[2], [3], [4]]. Early therapy is mandatory, including debridement of the penis, broad-spectrum antibiotics, repeated antiseptic dressings and skin grafting [2]. Early diagnosis by a strong index of suspicion ensures a favorable outcome [1,2]. As prevention is the key, this case highlights the importance of proper care and routine maintenance of condom catheters in order to avoid this devastating complication. Our work has been reported in line with the SCARE criteria [5].

## 2 Presentation of case

**Fig. 1:**
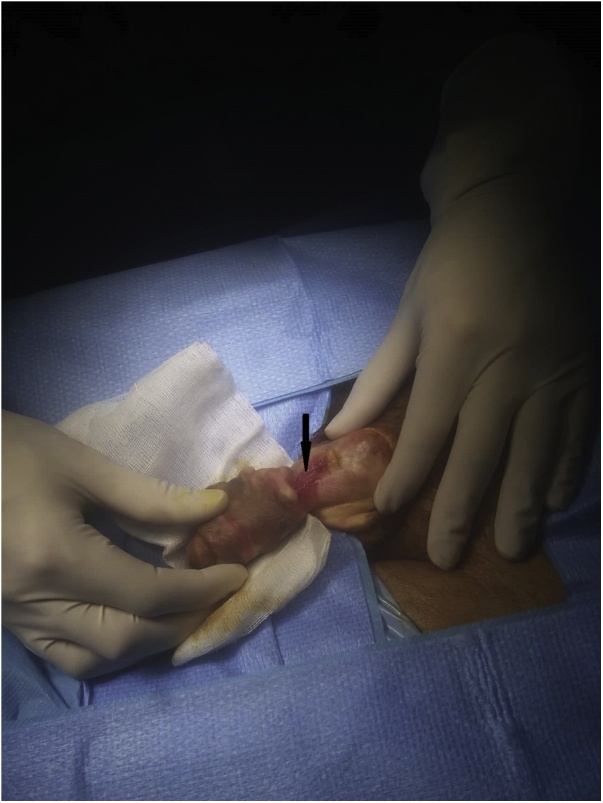
Dorsal view at time of operation. Partial loss of the urethra (arrow) and corpus spongiosum.

**Fig. 2:**
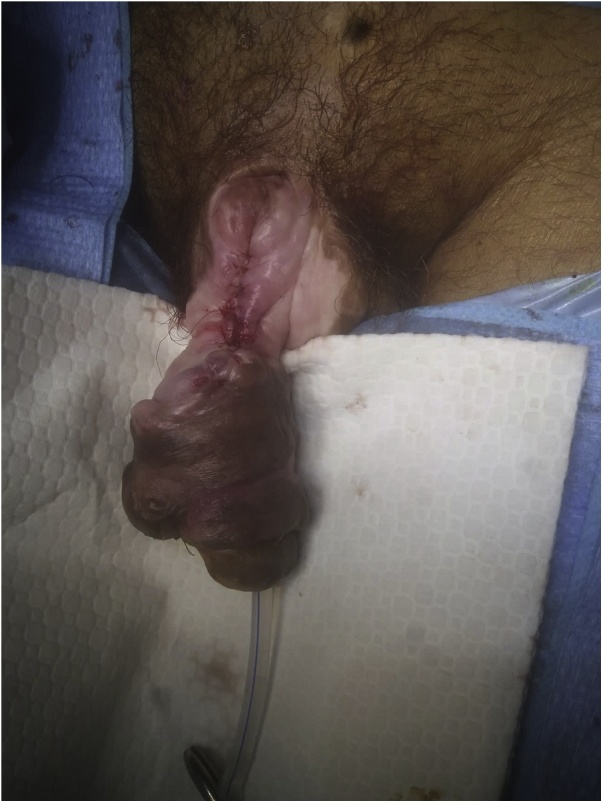
Post-operative appearance.

**Fig. 3:**
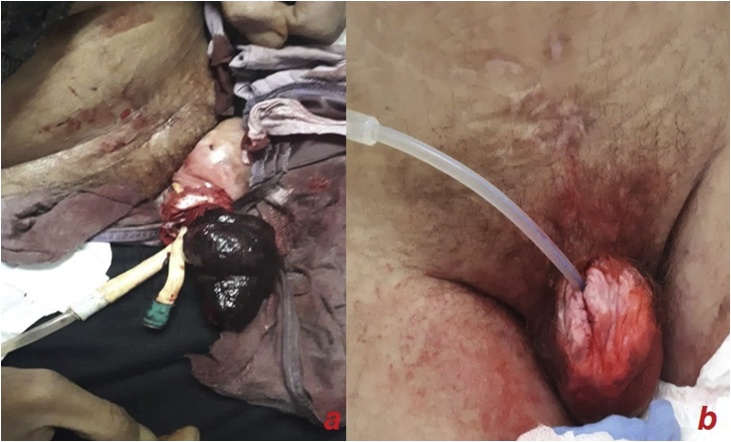
**a**. Necrosis of glans and distal two thirds of penis was with a partial amputation and section of the urethra. **b**. Partial penectomy.

A 58-year-old schizophrenic man, suffering from paraplegia due to post-traumatic spinal injury since seven years, had been using condom catheters for urinary incontinence. He was referred to our department for “penile injury”. The history of application and care of the catheter were not clear because the patient had not living relatives. Thorough interrogation revealed that he was placing a rubber band tightly around his penis to maintain the condom as its size was not appropriate. Physical examination showed a 4 cm defect of the proximal portion of the penis with a partial loss of both the corpus spongiosum and the urethra, delimited by a hard fibrous tissue (Fig. 1). A surgical repair was performed, after insertion a 18F silicon Foley catheter. It consisted in excision of necrotic tissues then covering the urethra using the surrounding tissue that was dissected and inverted, (Fig. 2). Due to the importance of the defect, we decided to repair the corpus spongiosum in a second intervention after improvement of tissue vitality. Unfortunately, the patient presented to the emergency department, 4 weeks after surgery for black discoloration of the penis. Interrogation found that he removed the Foley catheter 10 days after discharge, and that he put back the condom catheter fixed by a rubber band. On examination, the glans and the distal two thirds of the penis were totally necrotic with a partial amputation of the penis and a section of the urethra (Fig. 3a). Rest of the penile tissues and scrotum were normal and the patient had no fever. Laboratory findings, including white-blood-cells count and C-reactive-protein, were normal. Parenteral broad-spectrum antibiotics were administered and debridement under general anesthesia was performed, resulting in a partial penectomy (Fig. 3b). Local wound care was applied, and the patient was discharged uneventfully after a week. Follow-up after three months showed no signs of gangrene recurrence. Indwelling urethral catheterization was maintained and the patient was referred to the physical medicine and rehabilitation department for further management.

## 3 Discussion

Penile strangulation a rare condition firstly reported in 1755 by Gauthier [6]. Since then, approximately 60 cases have been reported in the world literature [[1], [2], [3], [4]]. The attachment of foreign bodies to the penis, leading to incarceration of that organ, has been accomplished using a variety of nonmetallic and metallic objects, including condom catheters and rubber bands [[1], [2], [3], [4],^7^,^8^].

Condom catheters are known as a safe tool to manage urinary incontinence in men population, providing an adequate care. Several sizes are available to accommodate anatomical variation, and they are designed to be worn 24/7 and changed every 24 or 48 h [9]. Many complications may occur, increasing in severity and frequency with long-term use, including urinary tract infection (40%) and skin injuries ranging from inflammation to gangrene due to strangulation of the penis [9]. However, gangrene is uncommon, probably because each corpus cavernosum has an individual artery, and the thickness of Buck’s fascia and corporeal tissue resists pressure on the deep vessels [9]. These complications are more common in debilitated patients with poor medical care or psychiatric disorders in developing countries [2]. In the reported case, three factors were involved: An oversized condom explaining the use of a rubber band, psychiatric disorder and a poor medical care in a patient with spinal cord injury (SCI). Indeed, after applying the rubber band on condom catheter, the tourniquet effect causes penile engorgement from the decrease in venous and lymphatic drainage. If the tourniquet effect continues, arterial flow is also compromised, resulting in ischemia and gangrene of the penis [2]. Lack of nociceptive feedback in SCI patients renders patients unaware of painful sensation due to the tourniquet effect of the catheter [2]. Condom catheter, through urine leakage, probably increases skin moisture around the penis [2]. Psychiatric disorders leading to compromised personal hygiene contribute to colonisation, and then rapid progression of the infection [1] as in our patient. The diagnosis of this condition is based on physical examination, with assessment of penile skin temperature, color, sensibility and pulsations distal to the constriction band. Color doppler analysis of the penis may help in identifying blood flow distal to the constriction band but is not mandatory [2]. In case of necrosis, debridement, which may result in a partial or a total penectomy if necessary, should be performed. If the diagnosis is made early, split thickness skin graft seems to provide good results [1,2]. Administrated antibiotics in the reported cases were mainly pippercillin-tazobactam, cefotaxime and metronidazole [[1], [2], [3]]. Prevention is the key to avoid these severe complications and it is based on simple measures. The appropriate size of condom should be selected and the penis should be inspected daily to ensure that the catheter is not placed too tightly. The applied condom catheter should be changed every 48 h. If glans or penile skin develops any sign of inflammation, immediate medical attention has to be sought as the penis can be salvaged by removing the catheter.

## 4 Conclusion

Condom catheter is frequently used to manage male urinary incontinence but it should not be used carelessly or overlooked as it can cause severe complications such as penile strangulation and penile gangrene. That’s why appropriate care is necessary, especially in debilitated and psychiatric populations. Increased awareness will facilitate prompt recourse to medical advice, when early signs of infection or gangrene are present.

## Funding

No source of funding.

## Ethical approval

Charles Nicolle Teaching Hospital ethic comitee, Tunis, Tunisia.

## Consent

Written informed consent was obtained from the patient for publication of this case report and accompanying images.

## Author contribution

Zaghbib S; concept or design, data collection, data analysis or interpretation, writing the paper.

Chakroun M; concept or design, data collection, data analysis or interpretation, writing the paper.

Saadi A; data collection, data analysis or interpretation.

Boussaffa H; data collection, data analysis or interpretation, writing the paper.

Bouzouita A; data collection.

Derouiche A; data collection

Ben Slama MR; data collection

Ayed H; data collection

Chebil M; writing the paper.

## Registration of research studies

This is no research study.

## Guarantor

Selim Zaghbib.

## Provenance and peer review

Not commissioned, externally peer-reviewed.

NO financial and personal relationships with other people or organisations that could inappropriately influence (bias) their work.

## Declaration of Competing Interest

NO financial and personal relationships with other people or organisations that could inappropriately influence (bias) their work.
